# Application of QuEChERS Method for Simultaneous Determination of Pesticide Residues and PAHs in Fresh Herbs

**DOI:** 10.1007/s00128-012-0951-x

**Published:** 2013-01-05

**Authors:** Anna Sadowska-Rociek, Magdalena Surma, Ewa Cieślik

**Affiliations:** Malopolska Centre of Food Monitoring, Faculty of Food Technology, University of Agriculture in Krakow, 122 Balicka street, 30-149 Krakow, Poland

**Keywords:** Herbs, Pesticides, PAHs, QuEChERS

## Abstract

The aim of this study was to evaluate the application of quick, easy, cheap, effective, rugged and safe method for simultaneous determination of polycyclic aromatic hydrocarbons and pesticide residues in fresh herbs. In the experiment two extraction solvents and standard types of sorbents were used. The extracts were analyzed using GC–SIM–MS. The results suggest that acetonitrile is more suitable extraction solvent giving more purified samples and better recovery values (71.6 %–116.9 %) with RSD lower than 15 % for most of the compounds. In real samples pesticides were identified in the samples of parsley, tarragon and lovage. In few samples the pesticide levels exceeded the MRL established by EU.

Herbs have been widely known for several purposes since ancient times. Some of them are willingly used for culinary purposes or a raw material for pharmaceutical products, cosmetics, and herbal medicinal products. Herbal spices are an essential element of food, improving the taste of food and giving them a distinctive flavour. Fresh spices are more valuable than dried, containing more vitamins and essential oils, and having a stronger aroma. Even though fresh herbal spices have many healthy benefits, they may be exposed to contaminants coming from the environment in which they are grown. Among them, residues of pesticides and polycyclic aromatic hydrocarbons (PAHs) are the most essential organic pollutants (Kosalec et al. 2009). Levels of PAHs in fresh plants are generally lower, but in plants that are often grown in close proximity to urban pollution sources, hence PAHs levels might be slightly higher (Hossain and Hoque 2011). Fifteen of these compounds were recognised as clearly mutagenic and carcinogenic by the Scientific Committee on Food, and benzo[a]pyrene and dibenzo[a,h]anthracene were reported to be the most dangerous. Additionally, an inappropriate use of plant protection products or too short withdrawal period may lead to its accumulation in herbal plants and in consequence human health problems. Pesticides are associated with a wide spectrum of hazards, from short-term impacts such as headaches and nausea to chronic impacts: cancer, reproductive harm, and endocrine disruption (U.S. EPA 2012).

Analysis of organic compounds in herbs encounters certain difficulties. Herbal plants have very complicated matrices with a wide range of biochemical composition and essential oils that interfere with received results. So far, conventional methods of sample preparation include liquid–liquid extraction with various solvents (hexane, acetone, dichloromethane) followed by suitable clean up: solid-phase extraction (SPE) on cartridges with alumina, florisil, silica, C_18_, PS-DVB or gel permeation chromatography (GPC). The final extract is usually analysed by gas or liquid chromatography coupled with one of variety detectors (Hajjo et al. 2007; Fenoll et al. 2008; Tuzimski 2011; Wang et al. 2011; Yin et al. 2011). Nevertheless, these traditional procedures are expensive, solvent intense and time-consuming and require advanced analytical equipment. The quick, easy, cheap, effective, rugged and safe (QuEChERS) method, developed originally for the determination of pesticide residues in food of plant origin, can be also an attractive alternative for analysis of organic contaminants such as mycotoxins, drugs, veterinary medicines, and finally, PAHs. As yet, the QuEChERS method has been applied occasionally to the study of pesticide residues in herbal plants in the works of Dai et al. (2011), Attallah et al. (2012) and Chen et al. (2012), and only a few analytical methods for the determination of organic pollutants in fresh herbs have been described in the recent literature (Slowik-Borowiec et al. 2012). Moreover, to our best knowledge, no researches concerning simultaneous determination of pesticides residues and PAHs in samples of fresh herbs have been conducted. Therefore, in this study we evaluated the possibility of the application of QuEChERS method for simultaneous determination of pesticide residues and PAHs in fresh herbs: basil, tarragon, sage, lovage, mint, parsley, rosemary, and oregano. The usefulness of the method was verified basing on the recovery ratio of analysed compounds.

## Materials and Methods

Acetonitrile, HPLC grade, ethyl acetate, for liquid chromatography LiChrosolv^®^ and formic acid, 98 %, p.a. were purchased from Merck KGaA, Germany. MgSO_4_ anhydrous p.a. and NaCl p.a., were purchased from Chempur SA, Poland. Na_3_Citrate dihydrate p.a., was obtained from Riedel-de Haen, Germany, Na_2_HCitrat sesquihydrate, 99 %, p.a., from Sigma-Aldrich Chemie GmbH, Germany. PSA SPE Bulk Sorbent and Carbon SPE Bulk Sorbent (GCB) derived from Agilent Technologies, USA. CLP Organochlorine Pesticide Mix, 531.1 Carbamate Mix and EPA 525 PAH Mix-B were obtained from Supelco, USA; Organophosphorous Pesticides Mix 1 (EPA 614) was purchased from Dr. Ehrenstorfer (Germany). Stock, intermediate and working standard solutions of pesticides and PAHs at concentration 2 μg mL^−1^ were prepared in hexane. Varian 4000 GC/MS (Varian, Inc., USA) system consisted of 3,800 GC and 4,000 Ion Trap MS detector was used to accomplish the GC–MS analyses. The injector was CP-1177 Split/Splitless Capillary Injector, with a temperature of 270°C, and a hand-injection volume of 1.0 μL. Each injection was performed in triplicate. Chromatographic separations were conducted using a Zebron MultiResidue-1 column (30 m × 0.25 mm × 0.25 μm; Phenomenex Inc., USA). The GC oven was operated with the following temperature program: 70–300°C (5°C min^−1^). Helium was used as the GC carrier gas at a flow rate of 1.0 mL min^−1^. The ion trap mass spectrometer was operated in the internal ionisation mode, scan from m/z 45 to 500. Analysis was conducted in the SIM mode, based on the use of one quantitative ions. Analysed compounds were identified according to their qualitative ions and retention times. Acquisition and processing data were collected using Varian Start Workstation software and NIST 2.0 library.

In first step we optimised the QuEChERS method using two extraction solvents (acetonitrile and ethyl acetate). The usefulness of the method was verified on the basis of the recovery ratio of analysed compounds in spiked samples. If it is assumed that investigated fresh herbs are in a similar biological family and have similar properties, then a similar composition of matrix can be used to represent all samples, such as we proposed in our previous work (Cieslik et al. 2011). For this reason, samples of lovage derived from organic farming with no pesticides and PAHs detected on previous occasions were used for recovery studies, and for the preparation of matrix-matched calibration.

Recovery studies involved three samples of fresh herbs being spiked with the standard solution of analysed compounds to the fortification level of 0.03 mg kg^−1^. The samples were spiked with mixture of standards, mixed and left to stand for 15 min at room temperature prior to extraction. The extraction process was conducted on all samples: a representative portion of fresh herb was cut, and macerated and homogenized in a blender. 10 g of sample was weighted into a 50 mL centrifuge tube. 5 mL of water and 10 mL of acetonitrile or ethyl acetate were added and the mixture was shaken vigorously for 1 min. After that 1 g Na_3_Citrate dihydrate, 0.5 g Na_2_HCitrat sesquihydrate, 1 g NaCl and 4 g MgSO_4_ were added, with the tube being shaken immediately after addition of the salt. Then each sample was shaken vigorously for 1 min., and centrifuged for 15 min at 8700 RCF. 6 mL of the supernatant was transferred into a PP 15 mL tube containing 0.15 g PSA, 0.05 g GCB and 0.9 g MgSO_4_. The tube were shaken for 2 min. and centrifuged for 5 min at 5000 RCF. A 4 mL amount from each of the extracts was transferred into a screw cup vial. The extracts were evaporated under a stream of N_2_ at a temperature of 40°C to dryness and then dissolved in 1 mL of hexane. The extracts were then analysed by GC–MS.

Blank samples were prepared in acetonitrile and ethyl acetate, respectively. It allowed estimating the signal of the plant matrix. Matrix-matched calibration standards at concentrations of between 0 and 400 ng mL^−1^ were prepared by adding known quantities of standard mixture solution to the corresponding blank sample extracts. In that case, plant extracts were prepared in acetonitrile, evaporated to dryness and the dry residues were dissolved in hexane.

Finally, we applied the optimised procedure to the determination of pesticide residues and PAHs in real samples of basil, tarragon, sage, lovage, mint, parsley, rosemary, and oregano.

## Results and Discussion

Calibration curves were constructed by plotting integrated peak areas against concentrations of compounds. Peak areas have been reduced by the area of the peaks of compounds derived from blank to eliminate the matrix effect. Therefore, calibration curves were calculated without y-intercept, which the high value could significantly affect the calculation of the results making them inaccurate. A sequence of least squares regression models were fitted and expressed by the Pearson correlation coefficient (r). No evidence for non-linearity was observed for all compounds in the concentration range (0–400 ng mL^−1^), and all values of r were higher than 0.99 except of carbofuran. The sensitivity of the calibration curves was much higher for the organochorine pesticides: DDT metabolites and derivatives, isomers of chlordane and hexachlorocyclohexane (HCH), but also for ethion, diazinon, and PAHs. The lowest sensitivity was obtained for carbamate pesticides, endrin and endrin aldehyde, endosulfan and endosulfan sulfate and also, surprisingly, for heptachlor. These differences are caused mainly by the compounds structure and composition that include the presence of the aromatic ring and non-polar properties. For polar compounds, such as carbamates, the method was less sensitive due to the type of applied GC column (non polar) and gas chromatography specificity that is less suitable technique for polar compounds. Similar results were obtained by comparing the limits of detection (LOD) and quantification (LOQ) of individual compounds. LOD and LOQ were estimated based on the signal of the background noise measured from the chromatograms of blank sample. LOD was calculated as three times higher than the level of noise, and the LOQ was equal to ten times of the noise level. LOQs for all compounds were lower than the 12 μg kg^−1^. The lowest levels were established for PAHs, DDE, methoxychlor, α-HCH, β-HCH, parathion, while the highest for carbamates, endosulfan and endosulfan sulfate and also for endrin aldehyde. The calibration data and LOD and LOQ values are shown in Table 1.

**Table 1:** Parameters of calibration curves, LOD, LOQ and recovery percentage of the investigated compounds

Compound	Correlation coefficient (r)	Calibration slope	LOD (μg kg^−1^)	LOQ (μg kg^−1^)	Recovery (%) ± RSD (%) in acetonitrile	Recovery (%) ± RSD (%) in ethyl acetate
Carbofuran	0.9890	52	3.3	10.0	99.0 ± 1.0	87.0 ± 4.0
Biphenylene	0.9956	257	0.3	1.0	98.3 ± 9.0	100.5 ± 7.0
Oxamyl	0.9988	42	4.0	12.0	103.0 ± 6.0	38.0 ± 17.0
1-Naphthol	0.9940	81	4.0	12.0	110.2 ± 13.0	97.5 ± 6.0
Methiocarb	0.9940	152	3.5	10.5	14.6 ± 25.0	78.7 ± 10.0
Fluorene	0.9915	166	0.3	0.9	130.8 ± 8.0	127.3 ± 5.0
α-HCH	0.9959	395	0.4	1.2	102.2 ± 14.8	125.1 ± 1.7
Diazinon	0.9980	618	0.8	2.5	101.7 ± 7.5	157.0 ± 1.6
β-HCH	0.9958	324	0.3	1.0	113.5 ± 12.0	162.6 ± 18.4
Disulfoton	0.9963	261	0.9	2.7	164.9 ± 4.0	5.6 ± 3.0
Anthracene	0.9962	266	0.3	1.0	145.5 ± 12.0	122.8 ± 6.0
Phenanthrene	0.9978	278	0.4	1.2	94.2 ± 10.0	87.2 ± 8.0
Lindane	0.9938	261	1.0	3.0	102.6 ± 1.9	110.7 ± 19.9
δ-HCH	0.9978	193	1.0	3.0	116.4 ± 7.7	77.0 ± 13.3
Heptachlor	0.9940	43	1.2	3.6	93.9 ± 3.4	148.4 ± 14.7
Methyl parathion	0.9928	156	1.3	3.9	163.5 ± 9.8	59.1 ± 8.0
Malathion	0.9943	167	1.2	3.6	101.7 ± 7.3	96.5 ± 17.7
Aldrin	0.9903	207	1.0	3.0	71.6 ± 4.6	37.1 ± 5.8
Parathion	0.9965	305	0.4	1.2	147.1 ± 20.5	208.3 ± 5.0
Heptachlor epoxide	0.9913	266	0.9	2.7	114.9 ± 0.5	21.7 ± 5.1
γ-chlordane	0.9946	427	0.8	2.5	77.4 ± 7.1	77.4 ± 26.2
α-chlordane	0.9958	389	0.8	2.5	72.0 ± 10.7	58.4 ± 10.9
Endosulfan	0.9982	96	3.2	9.6	116.9 ± 5.9	105.2 ± 33.3
Pyrene	0.9954	335	0.3	1.0	82.1 ± 10.0	104.8 ± 10.0
o,p′-DDE	0.9941	971	0.3	1.0	101.8 ± 3.7	210.1 ± 14.1
Dieldrin	0.9954	81	0.8	2.5	114.6 ± 7.3	34.3 ± 12.9
Endrin	0.9950	22	1.0	3.0	102.8 ± 5.8	166.2 ± 15.0
Ethion	0.9956	612	0.8	2.5	112.0 ± 6.0	185.7 ± 1.3
4,4′-DDD	0.9932	781	1.0	3.0	116.1 ± 13.6	86.5 ± 5.2
Endrin aldehyde	0.9926	72	3.3	9.9	69.5 ± 8.9	48.4 ± 16.8
4,4′-DDT	0.9920	204	1.1	3.3	108.1 ± 8.8	69.1 ± 15.1
Endosulfan sulfate	0.9934	26	3.4	10.2	77.6 ± 11.9	195.8 ± 19.4
Methoxychlor	0.9975	695	0.3	1.0	106.2 ± 7.7	135.4 ± 16.5
Chrysene	0.9965	312	0.4	1.2	10.5 ± 9.0	68.2 ± 12.0
Triphenylene	0.9988	359	0.5	1.5	24.1 ± 14.0	41.8 ± 13.0

Determination of organic compounds in fresh herbs involves specific problems with extraction, clean up and GC–MS analysis due to presence of chlorophyll and volatile oils in samples. The matrix can interfere with the analytes resulting in enhance or suppression of chromatographic peaks and ambiguity of identification. Therefore, we applied typical QuEChERS sorbents for matrix removal. Primary secondary amine (PSA) removes sugars, fatty and other acids and graphitised carbon black (GCB) is used for removal of pigments, such as chlorophyll. Additionally, we decided to use two extraction solvents in order to test their capabilities of simultaneous good analyte extraction from a sample and the least possible extraction of undesirable matrix components. Acetonitrile was the first solvent, used typically in QuEChERS method for pesticide extraction, and ethyl acetate was the second tested solvent, chosen for the fact that it is applied for the extraction of PAHs. The choice of more suitable solvent was based on recovery ratio value of spiked samples but the visual impressions (sample colour, its transparency, and the content of impurities) of obtained extracts were also taken into consideration.

The extracts of herbal plants were more saturated and colourful in case of extraction with ethyl acetate than after the use of acetonitrile. Comparing the chromatograms of the investigated samples, peaks of analytes in ethyl acetate extracts were characterized by a stronger signal than in the acetonitrile extracts.

**Fig. 1:**
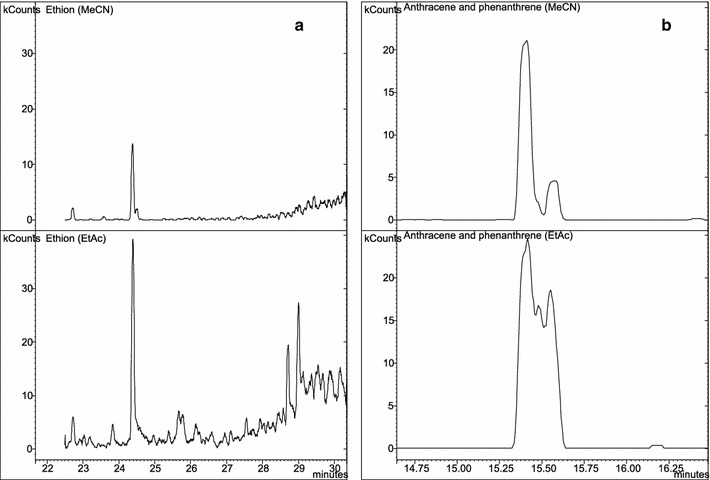
**a** Peak of ethion in samples extracted with acetonitrile (MeCN) and ethyl acetate (EtAc). **b** Peaks of anthracene and phenanthrene in samples extracted with acetonitrile (MeCN) and ethyl acetate (EtAc)

For some compounds, especially for heavier PAHs (pyrene, chrysene, triphenylene), this signal from ethyl acetate extracts was several times higher, which improved the recovery of the compounds. However, for the other compounds, e.g. ethion, an exceptionally strong enhance of the signal was observed (Fig. 1a). This fact can be explained as the influence of matrix components, especially the volatile oils, having the same qualifier ions and interacting with the analytes. Ethyl acetate, in contrast to acetonitrile, shows a greater tendency to extract volatile oils. This phenomenon was also confirmed during the final preparation of the samples by evaporation the extracts to dryness. After evaporation, in the residues of the samples extracted with ethyl acetate there were much more impurities and oils with characteristic, herbal odour than in the residues of the samples after extraction with acetonitrile. Use of ethyl acetate resulted also in the extraction of contaminants from the sample and incomplete separation of the peaks (e.g. the appearance of the additional peak between phenanthrene and anthracene, Fig. 1b). Therefore, it was decided not to use the matrix-matched calibration using blank samples prepared in ethyl acetate.

Recovery studies were conducted after fortification to the levels of 0.03 mg kg^−1^. For both type of solvents, satisfactory recovery values of pesticides (70 %–120 %) were obtained for carbofuran, naphthol, lindane, δ-hexahlorocyclohexane, malathion, γ-chlordane, endosulfan, DDD. For the rest of examined pesticides the recoveries were better for the samples extracted with acetonitrile, except of methiocarb, for which the recovery was better for the samples extracted with ethyl acetate. In the group PAHs, the best recovery was noticed for biphenylene, phenanthrene and pyrene. For fluorene and anthracene the recovery significantly exceeded the limit of 120 %, which was presumably caused by the influence of the plant matrix. For the rest of the compounds the recovery ratio did not exceed 30 %. In most cases the recovery ratio of PAHs was slightly better in the samples where ethyl acetate was used for the extraction. Low values of the recovery ratio in case of heavy PAHs were probably influenced by the use of sorbents GCB that might have removed some compounds with planar structure, from the samples. The repeatability of recovery values, expressed as the relative standard deviation (RSD) of the spiked sample concentrations, was lower than 15 % for carbofuran, diazinon, ethion, heptachlor epoxide, biphenylene, α-chlordane. Repeatability was more varied in case of ethyl acetate extraction (1.3 %–33.3 %) and usually higher than for the samples extracted with acetonitrile. RSD lower than 5 % was discovered for carbofuran, diazinon, and heptachlor epoxide. For certain compounds RSD were higher than 20 % (methiocarb in acetonitrile extract, γ-chlordane and endosulfan in ethyl acetate extract).

The achieved results of recovery indicate that acetonitrile is a more suitable solvent for the extraction of pesticide residues, while ethyl acetate has a greater ability to extract PAHs. However, considering the appearance of extracts, the content of interacting components derived from the matrix, especially essential oils, it was concluded that acetonitrile is a better solvent for the simultaneous extraction of residues of pesticides and PAHs in samples of fresh spices. For this reason, for further studies of real samples, it was decided to use acetonitrile for the extraction.

Table 2 summarizes the results in real samples of analysed fresh herbs. The samples (n = 10) were purchased on a local market. Pesticide residues were found in most investigated plants except of oregano, and the greatest number of pesticides was identified in the samples of parsley, tarragon and lovage. The organochlorine pesticides were the group that was identified most frequently, but its levels were below or very close to the limit of quantification or to the MRL values established by EU. In few samples of parsley, sage and rosemary the pesticide levels (HCH isomers, endosulfan and carbofuran) slightly exceeded MRLs (bolded in Table 2). No PAHs residues were detected in analysed samples.

**Table 2:** Results of real samples analysis

Compounds	Residues (mg kg^−1^)							
	Sage	Basil	Tarragon	Lovage	Mint	Oregano	Parsley	Rosemary
Carbofuran				**0.060**	**0.067**			**0.078**
α-HCH				0.002			**0.006**	
β-HCH			0.001			<LOQ	**0.003**	
Lindane				0.010			0.007	
δ-HCH	0.003		0.004				**0.007**	
Carbaryl		<LOQ	<LOQ		<LOQ		<LOQ	
γ-Chlordane				<LOQ			<LOQ	
Endosulfan	0.039	0.051	**0.048**	0.017				**0.017**
Dieldrin	**0.011**					<LOQ	0.009	
Ethion		0.007		<LOQ				
4,4′-DDD		0.003					0.019	
Endrin aldehyde			**0.014**	<LOQ	0.018		0.029	
Endosulfan sulfate			**0.042**	0.015				**0.040**
Methoxychlor			0.009	0.005				

In general, a new approach for simultaneous analysis of pesticide residues and PAHs in fresh herbs has been proposed, using the QuEChERS method. The results revealed that the QuEChERS method could be successfully applied for the determination of selected compounds in herbs. However, fresh herbal plants are the matrices that require careful sample prep to ensure valid results, and the presence of essential oils is the main problem in the analysis.
